# Osmoprotectants in Hybrid Liposome/HPMC Systems as Potential Glaucoma Treatment

**DOI:** 10.3390/polym11060929

**Published:** 2019-05-28

**Authors:** Miguel Gómez-Ballesteros, José Javier López-Cano, Irene Bravo-Osuna, Rocío Herrero-Vanrell, Irene Teresa Molina-Martínez

**Affiliations:** 1 Complutense University, Innovation, Therapy and Pharmaceutical Development in Ophthalmology (InnOftal) Research Group, UCM 920415, Department of Pharmaceutics and Food Technology, Faculty of Pharmacy, Plaza Ramón y Cajal s/n, Madrid 28040, Spain; mgballesteros@farm.ucm.es (M.G.-B.); josejavl@ucm.es (J.J.L.-C.); ibravo@ucm.es (I.B.-O.); 2 Ocular Pathology National Net (OFTARED) of the Institute of Health Carlos III, Health Research Institute of the San Carlos Clinical Hospital (IdISSC), Madrid 28040, Spain

**Keywords:** liposomes, HPMC, glaucoma, acetazolamide, ocular tolerance, intraocular pressure

## Abstract

The combination of acetazolamide-loaded nano-liposomes and Hydroxypropyl methylcellulose (HPMC) with similar components to the preocular tear film in an osmoprotectant media (trehalose and erythritol) is proposed as a novel strategy to increase the ocular bioavailability of poorly soluble drugs. Ophthalmic formulations based on acetazolamide-loaded liposomes, dispersed in the osmoprotectant solution (ACZ-LP) or in combination with HPMC (ACZ-LP-P) were characterized and in vivo evaluated. The pH and tonicity of both formulations resulted in physiological ranges. The inclusion of HPMC produced an increment in viscosity (from 0.9 to 4.7 mPa·s. 64.9 ± 2.6% of acetazolamide initially included in the formulation was retained in vesicles. In both formulations, a similar onset time (1 h) and effective time periods were observed (7 h) after a single instillation (25 μL) in normotensive rabbits’ eyes. The AUC_0–8h_ of the ACZ-LP-P was 1.5-fold higher than of ACZ-LP (*p* < 0.001) and the maximum hypotensive effect resulted in 1.4-fold higher (*p* < 0.001). In addition, the formulation of ACZ in the hybrid liposome/HPMC system produced a 30.25-folds total increment in ocular bioavailability, compared with the drug solution. Excellent tolerance in rabbits’ eyes was confirmed during the study.

## 1. Introduction

Glaucoma is considered the second leading cause of blindness in the world after cataracts and one of the main causes of irreversible blindness. This disease is expected to affect 111.8 million patients in 2040 [1,2]. The aetiology of the disease is not completely clear. Glaucoma is considered as a group of chronic eye neuropathies characterized by the non-reversible degeneration of retinal ganglion cells, whose axons form the optic nerve [3]. In most cases, glaucoma is associated with a high and continuous elevation of intraocular pressure (IOP) due to an accumulation of aqueous humour in the anterior segment of the eye owing to different causes. This increment in IOP is spread to the posterior segment of the eye and generates injury in the retina and optic nerve, and also compromises the blood flow in this area, contributing to the damage [4]. For this reason, the topical administration of antihypertensive drugs is the first therapeutic step in clinical practice when IOP is increased [5]. These substances can reduce aqueous humour production and/or promote its drainage. However, the main restriction of this therapy is the lack of patients’ compliance, due to the need for frequent applications and the appearance of severe ocular surface discomfort after chronic treatments. This, joined to the fact that glaucoma is asymptomatic until the last stages of the disease, explains why almost 60% of patients do not fulfill with the prescribed treatment [6,7]. The use of eye-drop formulations able to improve the ocular bioavailability of antihypertensive drugs might reduce the number of applications per day. In addition, if the preparation includes components that can reduce the ocular surface alterations the long-term patient compliance would increase.

Acetazolamide (ACZ) is a carbonic anhydrase inhibitor with a potent specific effect in reducing aqueous humour production and, therefore, intraocular pressure [8,9,10,11]. According to the biopharmaceutical classification system (BCS), acetazolamide is a class IV drug (low solubility and low permeability) so, unfortunately, its topical instillation in simple solution is not effective [12,13,14]. Several technological approaches have been recently investigated to increase its solubility and/or its retention time on the ocular surface and, as a consequence, its ocular bioavailability. Some of these include the use of niosomes [15], cyclodextrins [16], dendrimers [17], nanoparticles or liposomes [18].

Niosomes are prepared from amphiphilic mixtures in aqueous media that create bilayer vesicles. Some authors argue that niosomes are in between liposomes and microemulsions, since they can entrap hydrophilic and poor water-soluble substances (an inherited characteristic from liposomes). In addition, they have been described to enhance corneal penetration due to their surfactant properties, similar to those present in the microemulsions [19]. Despite the fact that niosomes exhibit an adequate corneal permeability, they were proven to be toxic for corneal cells, particularly those charged positively [20]. In spite of these findings, niosomes formulations could be improved and considered a potential therapeutic tool for the treatment of ocular diseases. 

Another interesting approach involves the use of cyclodextrins, which are very versatile compounds composed of oligosaccharides creating a cyclic structure [21]. They are able to form interesting systems that present good properties for drug delivery and particularly for the treatment of ocular pathologies. In fact, a recent pilot study demonstrated that eye drops containing γ-cyclodextrin based-nanoparticles loaded with dexamethasone presented good tolerance and high efficacy ratios in patients suffering from diabetic macular edema [22].

Dendrimers are super branched-like structures ranging from 1–200 nm able to transport active substances. Due to their particular structure, the interactions between dendrimers and corneal tissue could improve ocular bioavailability [19]. Besides all these, dendrimers could avoid some problems such as blurred vision, commonly described with the use of bioadhesive substances.

Another interesting approach regarding ocular drug delivery strategies is the use of solid lipid nanoparticles (SLNs) and nanostructured lipid carriers (NLCs). Apart from the well-known advantage of encapsulating lipophilic drugs, they can undergo autoclave sterilization and low toxicity. They also have in common with the previous systems mentioned before that could entail an efficient ocular drug delivery system due to the enhancement of corneal penetration [23].

The use of polymeric nanoparticles such as polysaccharide like chitosan, gelatin or poly lactic-co-glycolic acid (PLGA) based nanoparticles are extended in the development of ocular drug delivery systems. They protect the active ingredients in the inner core from external degradations and allow the controlled release of the drug. These certain characteristics are particularly interesting with respect to topical ocular administration due to the presence of enzymes that could inactivate the active substances. It also makes the nanoparticles especially resistant to shear forces present in the ocular surfaces allowing them to maintain their structure [24].

Nanosuspensions work as an effective alternative poor soluble substances such as those that tend to form crystal. Furthermore, these carriers do not create discomfort or swelling after their application, therefore they are considered as an inert carrier for ocular drug delivery [25]. Nanoemulsions have interesting properties for delivering drugs topically in ophthalmology. One excellent characteristic is their good spreadability and their stability [26]. 

Liposomes are one of the most explored due to their important advantages such as high ocular compatibility, lack of immunogenicity and low toxicity. Furthermore, liposomes can be loaded with poorly soluble drugs that have low bioavailability in suspensions or solutions forms, e.g., idoxuridine for acute and chronic herpetic keratitis, penicillin G, Indoxole or some steroids such as triamcinolone acetonide [27]. In addition, the simplicity of the formulations and the versatility of their physical characteristics make liposomes very suitable for the ocular administration of drugs [28]. 

Another interesting strategy to increase the ocular residence time of active compounds is the inclusion of polymers in the eye-drops formulations. They contribute to forming a viscous continuous layer on the ocular surface able to partially protect the drug against lacrimal drainage, extending the ocular residence time [29,30]. In fact, polymers such as carboxymethylcellulose, hydroxypropyl methylcellulose (HPMC), hyaluronic acid or xanthan gum have been employed with this aim [19,30,31]. All of them have shown also good in vitro and in vivo tolerance. The bioavailability of drugs applied topically can be increased by extending the residence time of the ophthalmic formulation. Moreover, the use of polymers together with the inclusion of liposomes and other nanosystems makes of this approach ideal to reach higher effectiveness, therefore managing more successfully the treatment of ophthalmic diseases [32].

It has been widely recognized that hyperosmolarity may play a crucial role by worsening the pro-inflammatory responses in the ocular surface [33]. Osmoprotectants are osmolytes which act as protecting cells and microorganisms from osmotic stress. The accumulation of osmolytes result in a maintenance of the cell fluid balance and cell volume, and hence keeping the equilibrium balanced with the external environment [34]. There are several osmoprotective substances that have demonstrated protecting properties such as L-carnitine, erythritol or trehalose among others [35]. The use of these osmoprotective substances can be considered as a potential therapeutic tool to tackle the harmful effects of different anti-glaucoma drugs such as dry eye or ocular discomfort.

This experimental work has been conducted to develop a novel eye-drop formulation useful in the treatment of glaucoma. To this, the mentioned technological strategies have been combined. In a first step, acetazolamide loaded liposomes were prepared in an osmoprotectant aqueous solution containing borates, trehalose and erythritol, characterized and in vivo evaluated in terms of hypotensive efficacy. Furthermore, the developed formulation was subsequently combined with HPMC to create a new hybrid liposome/polymer system in an osmoprotectant media able to increase the acetazolamide bioavailability and therefore its hypotensive activity after ocular topical administration, trying to preserve at the same time the ocular surface and the functionality of the precorneal film. To this, both efficacy and tolerance in vivo studies of the hybrid osmoprotectants/liposome/HPMC formulation were performed. To our knowledge, this is the first time in which a hybrid system (liposomes/polymer) exclusively composed by components similar to those present in the precorneal film, and with osmoprotectants, are prepared and in vivo tested for glaucoma treatment.

## 2. Materials and Methods 

### 2.1. Materials 

Phosphatidylcholine (PC) (Phospholipon 90G^®^) was obtained from Lipoid GmbH (Ludwigshafen, Germany). Trehalose and erythritol were purchased from Cymit Química S.L. (Barcelona, Spain). HPMC was supplied by Abarán materias primas (Madrid, Spain). Cholesterol (Ch) and Vitamin E were purchased from Sigma-Aldrich Chemical Co. (Madrid, Spain). Acetazolamide (ACZ) was supplied by Fagron Ibérica S.A.U (Barcelona, Spain). All solvents and other reagents were obtained from Panreac Química S.A. (Madrid, Spain) and used as received. 

### 2.2. Animals 

Male New Zealand white rabbits (San Bernardo Farm, Navarra, Spain), weighing 3–3.5 kg, normotensive, were used for in vivo experiments. They were kept in individual boxes with food and water *ad libitum* under controlled light/dark cycles (12/12 h) and in a room with controlled temperature and humidity (22 °C and 50% relative humidity). The animals were handled following the European Union regulations for the use of animals in research and the ARVO (Association for Research in Vision and Ophthalmology) Statement for the Use of Animals in Ophthalmic Vision Research [36], European Communities Council Directive (86/609/EEC) and Spanish Regulation of Experimental Studies with Animals (RD 53/2013, February 1; Ref PROEX 316/16, January 25 2017). 

### 2.3. HPLC Quantification of Acetazolamide

Acetazolamide quantification was carried out using a Gilson HPLC instrument (Middleton, WI, USA), a 305 solvent delivery pump, a 118 UV–vis detector and UniPointTM^®^ controller software. The injector was equipped with a 20 μL loop 7125 Rheodyne (Middleton, WI, USA). The chromatographic separation was achieved by a reversed phase protocol with a Tracer Excel ODSA column (25 cm × 4 mm, 5 μm particle size) (Teknokroma, Barcelona, Spain). The mobile phase was a mixture of sodium acetate and ultrapure (milliQ) water (1:5). The flow rate was set at 1mL/min and the eluent was monitored at 245 nm. The quantification of acetazolamide in the liposome was performed after lyophilization and subsequent dissolution in ethanol. The method was validated in terms of linearity, accuracy and precision in the concentration range of 1–10 µg/mL.

### 2.4. Preparation of Acetazolamide Liposomal Formulations

Liposomes (LP) were prepared by the solvent evaporation technique as previously described [37]. To this, 15 mg of acetazolamide was dissolved in 20 mL of ethanol by stirring for 24 h. PC, Ch and vitamin E were then added. The ratio of Pc:Ch:Vit-E:ACZ components in the organic solution was 8:1:0.08:0.3 respectively. The solvent was evaporated under reduced pressure (50 hPa) on a rotary evaporator (Buchi R-205, Mass Analytical S.A., Barcelona, Spain) at 33 °C for 60 min. The film formed was then hydrated with dispersion solution of borates, trehalose and erythritol (named hereinafter “base vehicle”, BV). The composition of this aqueous solution was as follows: 8.38‰ H_3_BO_3_, 0.755‰ Na_2_B_4_O_7_, 29.8‰ trehalose and 6.1‰ erythritol. The lipid vesicles were extruded through a size-controlled 0.2 μm pore size polycarbonate membrane (Spectra/Por^®^ dialysis membrane, MWCO 3500, Spectrum Laboratories, Iberlabo, Madrid, Spain) for 10 cycles under nitrogen pressure (1,379 MPa) to obtain lipid vesicles with a homogenize size distribution. The final formulations were prepared by dilution 1:2 with the corresponding solutions: for the ACZ liposomal formulation (ACZ-LP) the dilution was performed with the base vehicle (BV). For the liposomal formulation included in the HPMC (ACZ-LP-P) the dilution was performed with a solution of 0.6% HPMC prepared in the base vehicle. Final PC and ACZ concentrations in the final dispersions were 20 mg/mL and 0.7 mg/mL, respectively. The final composition of the two liposomal formulations prepared is described in Table 1.

All preparation steps were performed under aseptic conditions. Base vehicle and polymer solution were sterilized by autoclaving (Autester ST DRY PV-111, Selecta, Barcelona, Spain) and the final formulations underwent sterilizing filtration (0.2 µm). For comparative purposes, a saturated solution of ACZ in VB was prepared by stirred overnight to ensure total dissolution, with a final concentration of 0.7 mg/mL.

### 2.5. Liposomal Formulations Characterization 

#### 2.5.1. Mean Particle Size and Size Distribution

Mean particle size and particle size distribution of ACZ-LP formulation was measured by dynamic light scattering using a particle analyzer (Microtrac^®^ S3500 Series Particle Size Analyzer (Montgomeryville, PA, USA) at room temperature in milliQ^®^ water. 

#### 2.5.2. pH Determination

A pH-meter (pH-meter (model 230, Mettler, Barcelona, Spain) equipped with a microelectrode (InLab, Mettler, Madrid, Spain) was used to measure the pH of the formulations. Data were recovered in triplicate at room temperature.

#### 2.5.3. Osmolarity Analysis

Osmolarity was analyzed by vapor pressure osmometer (model k-7000: Knauer) at 33 °C (ocular surface temperature) [38]. The apparatus was previously calibrated with 400 mOsm/L NaCl solution. 

#### 2.5.4. Viscosity Evaluation

Viscosity was evaluated with a rheometer (Rheostress R1, Haake) using a parallel plate system (60 mm diameter and 0.5 mm separation). Viscosity was measured when the steady sate was reached with shear rates increasing from 0 to 1000 s^−1^ in 20 steps. The determination was performed in triplicate at 33 °C. 

#### 2.5.5. Surface Tension Measurement

Surface tension was measured with a tensiometer (K-11, Kruss) using the Wilhelmy plate method. Before each measurement, the tensiometer was calibrated with MilliQ water (72.0 ± 1 mN/m). The time required for equilibration of the formulations was set to 3 min. The formulations were assayed in triplicate. 

#### 2.5.6. Entrapment Efficiency Quantification

The drug loading was determined by an ultra-filtration method [39]. Briefly, 500 μL of ACZ loaded-liposomal dispersion was placed into an Amicon Ultra 4 centrifugal filter unit with a nominal molecular weight limit of 10 KDa (Merck Millipore Ltd., Darmstadt, Germany). The membranes were previously rinsed with Mili-Q^®^ water, immediately filled with the dispersions and centrifuged at 5300× *g* for 60 min (Hettich Universal 32). The amount of free ACZ was analyzed by HPLC as previously described. The entrapped drug was obtained by subtracting the amount of free ACZ from the total drug incorporated in 500 μL of ACZ loaded-liposomes. The entrapment efficiency (EE) was calculated using the following equation (Equation (1)).

(1) $$\mathrm{EE}\%=\frac{\left(\mathrm{Total}\;\mathrm{amount}\;\mathrm{of}\;\mathrm{ACZ}-\mathrm{Free}\;\mathrm{amount}\;\mathrm{of}\;\mathrm{ACZ}\right)}{\mathrm{Total}\;\mathrm{amount}\;\mathrm{of}\;\mathrm{ACZ}}\;\times \;100$$

### 2.6. Intraocular Pressure Measurements

Efficacy studies were performed in normotensive rabbits. 25 μL of the corresponding formulation was applied to both eyes of the same rabbit. IOP measurements were performed hourly over a period of 8 h with a Tonovet rebound tonometer (Tiolat, Helsinki, Finland). 

The efficacy in vivo studies were carried out in 2 stages according to designs of cross-tests in which several variables were evaluated: the animal, the period and the treatment. In the first stage, 3 treatments were studied in three consecutive periods (design crossover 3 × 3) and in the second stage 2 treatments were evaluated (design crossover 2 × 2). Six animals were used for each treatment (12 eyes) in both cases. The minimum wash time between consecutive treatments was 48 h. Furthermore, a minimum period of 72 h was established between the two stages of the study. 

The assay treatments in the first stage were: (i) ACZ-LP formulation, (ii) base vehicle (BV) and (iii) 0.7 mg/mL ACZ solution in vehicle base, ACZ-VB.

In the second stage, the treatment corresponded to liposomal formulations: (i) liposomes alone ACZ-LP and (ii) hybrid system formed by liposomes included in the polymer solution ACZ-LP-P.

The hypotensive activity of each treatment was defined in terms of IOP pressure reduction (ΔIOP). This data was calculated using as reference the IOP basal (100%) data determined 30 min before and immediately before instillation of the formulation or base vehicle. The maximum percentage of IOP reduction (ΔIOP_max_) and the area under the ΔIOP (%)-time curve from 0 to 8 h (estimated by the trapezoidal rule, AUC(_0–8h_) were calculated for the different formulations and stages. Other parameters such as the maximum intraocular pressure (ΔIOP_max_), the onset time of hypotensive effect (*t*_onset_) and the effective time period were also evaluated.

### 2.7. Tolerance Studies 

The ocular tolerance study of the two liposomal formulations, acetazolamide-loaded liposomes alone with the osmoprotectant solution (ACZ-LP) or in combination with HPMC solution (ACZ-LP-P) was performed by instillation of 25 µL of the formulation each 30 min for 6 h onto the right eye in six male New Zealand albino rabbits. The contralateral eye received the same volume of saline solution (control). The examination of the eyes was performed by specular microscopy before instillation, just after instillation, and at three, six and twenty-four hours post instillation. A slit lamp (SL-8Z, Topcon, Barcelona, Spain) was used to evaluate clinical signs (pupillary reflex, pupil size, superior and inferior eyelids, presence of redness, blepharitis and blepharospasm, tear charge, exudates, fluorescein tear film breakup time (TBUT), redness of bulbar, limbal, and tarsal conjunctival surfaces, inflammation of nictitating membrane, and transparency of the cornea). Ocular signs were graded using a modification of the scoring system established in the guidelines of the Organization of Cooperation and Development in 2002 [40] and the protocols described by Enriquez et al. [41].

### 2.8. Statistical Analysis

Data were expressed as the means ± standard errors of the mean. Statistical differences were evaluated by analysis of variance (ANOVA). *P*-values less than 0.05 were considered significant. For efficacy studies, a comparison of the confidence interval was performed for several activity parameters: maximal IOP reduction and area under the curve of the IOP variation versus time plot. The treatments were considered significant when the two-sided 95% confidence interval for the difference between the means of the selected parameters excluded zero [42]. Stat graphics centurion 18 analysis software was used to perform the statistical analysis.

## 3. Results

### 3.1. Liposomal Formulation Characterization

**Figure 1:**
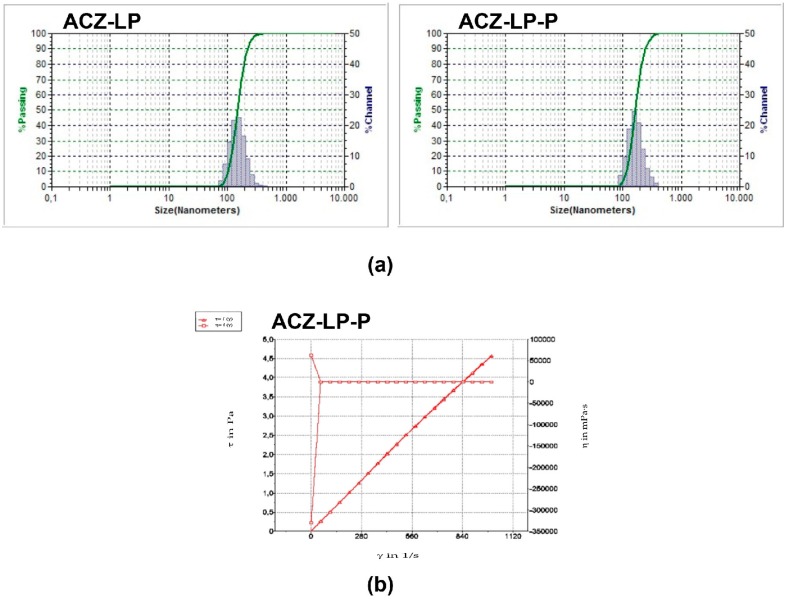
(**a**) Particle size distribution and (**b**) rheological behaviour of formulations composed by acetazolamide-loaded liposomes alone (ACZ-LP) or in combination with HPMC (ACZ-LP-P).

The physicochemical data obtained from both liposomal formulations, the acetazolamide-loaded liposome in osmoprotectant solution (ACZ-LP) and its combination with HPMC (ACZ-LP-P) are shown in Table 2 and Figure 1.

The mean particle size in both cases resulted similar and lower than 200 nm. Unimodal distribution was always observed (Figure 1a). pH of both formulations falls within the range 6.5–9 avoiding discomfort at the time of application [43,44]. The surface tension of ACZ-LP was significantly lower than that ACZ-LP-P (*p* < 0.05). The osmolarity of the formulations was within the range of isotonicity [45]. In both liposomal formulations, the viscosity values are similar to those of human tear (0.3 to 8.3 mPa·s) [46,47], however, the addition of HPMC significantly increases this parameter from 0.9 to 4.7 mPa·s maintaining a Newtonian behavior (Figure 1b). 

According to drug loading calculations, 64.9 ± 2.6% of acetazolamide initially included in the formulation was retained in liposomal vesicles, being the rest of the drug present in the aqueous solution surrounding them.

### 3.2. Hypotensive Activity of Liposomal Formulations on IOP in Rabbits

#### 3.2.1. Stage 1

**Figure 2:**
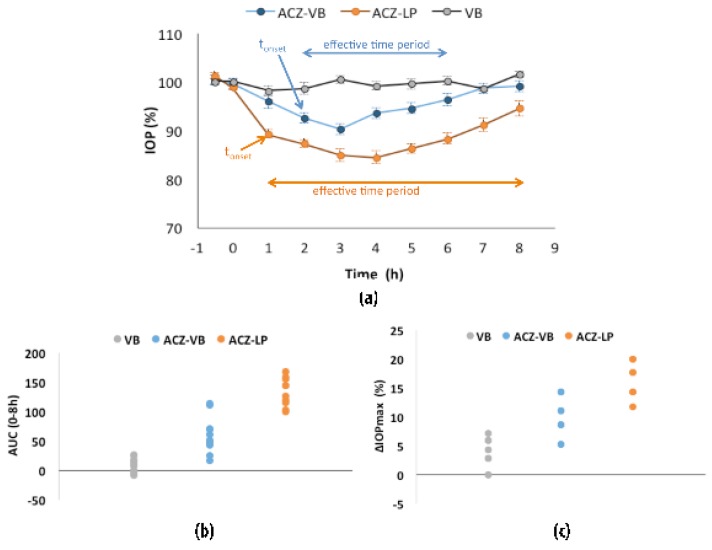
Stage one: (**a**) Intraocular pressure (IOP, %) mean after instillation of 25 µL of ACZ-VB (0.7 µg/mL acetazolamide in vehicle base), ACZ-LP liposomal formulation (0.7 mg/mL acetazolamide and 20 mg/mL liposomes expressed in concentration of PC in vehicle base) and VB (vehicle base used as control). (**b**) Area under curve of the ΔIOP (%) versus time (h) from 0 to 8 h—AUC _(0–8h)_. (**c**) Maximal IOP reduction (ΔIOP_max_, %). The results are expressed as the mean ± standard error.

In this first stage, the efficacy of the acetazolamide-loaded liposomes formulation was tested and compared to the solution of the drug in vehicle base and vehicle base alone. Data are presented in Figure 2 as well as in Table 3 and Table 4. According to the ANOVA analysis performed, there were no significant differences between time period, animal and eye (right or left) of the same animal for the parameters studied (AUC_0–8h_ and ΔIOP_max_).

As expected, the control vehicle base (VB) alone had no hypotensive effect. On the contrary, when a simple solution of ACZ in this vehicle (ACZ-VB) was instilled in rabbit eyes, the onset of hypotension was evident at 2 h post instillation and lasted for a total period of four hours (Figure 1a). When the same amount of drug was formulated in liposomes (ACZ-LP) the hypotensive onset was more rapid and already observed 1h post instillation. Furthermore, the hypotensive effect was maintained until the end of the study (a total of 7h) (*p* < 0.05). ANOVA analysis performed to compare the efficacy of ACZ-VB versus ACZ-LP in terms of ΔIOP_max_, and the AUC_0–8h_ revealed significant differences between treatments: the formulation of ACZ in liposomes led to a maximum hypotensive effect of 16.6%, significantly greater than the value achieved by ACZ-VB, 10%. Furthermore, The AUC_0–8h_ of ACZ-LP 137.4% h was 2.3-folds higher than that of ACZ-VB. The mean values of the ΔIOP_max_, and the AUC_0–8h_ and their 90% confidence limits are listed in Table 4.

#### 3.2.2. Stage 2

**Figure 3:**
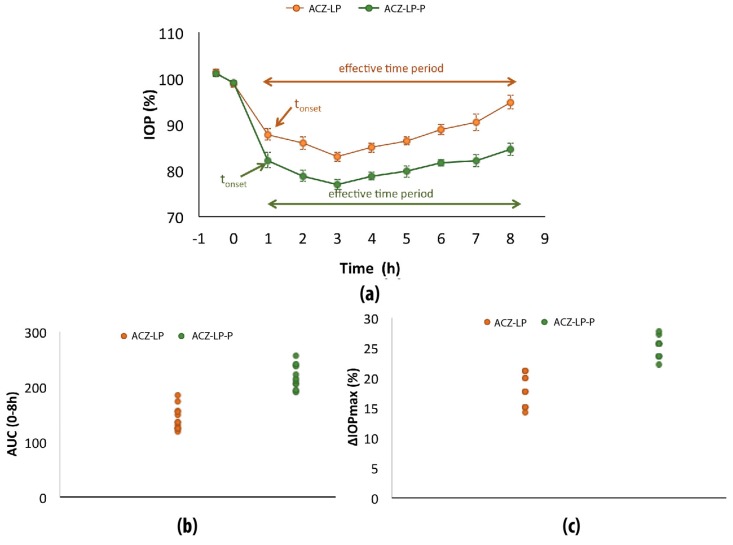
Stage two: (**a**) Intraocular pressure (IOP, %) mean after instillation of 25 µL of ACZ-LP liposomal formulation (0.7 mg/mL acetazolamide and 20 mg/mL liposomes expressed in concentration of PC in vehicle base) and ACZ-LP-P (0.7 mg/mL acetazolamide, 20 mg/mL liposomes expressed in concentration of PC and 0.3% HPMC in vehicle base). (**b**) Area under curve of the ΔIOP (%) versus time (h) from 0 to 8 h—AUC_(0–8h)_. (**c**) Maximal IOP reduction (ΔIOP_max_, %). The results are expressed as the mean ± standard error.

In this second stage, the efficacy of the acetazolamide-loaded liposomes in the osmoprotectant solution (ACZ-LP) or including also HPMC (ACZ-LP-P) was compared. Data are presented in Figure 3, Table 5 and Table 6. In both cases, similar onset time and effective time periods were observed. However, the combination of the ACZ-loaded liposomes with HPMC resulted in a more pronounced effect, as was suggested in Figure 2 and confirmed by ANOVA analysis. The mean values of the ΔIOP_max_, and the AUC_0–8h_ and their 90% confidence limits are listed in Table 5.

Effectively, the AUC_0–8h_ of the ACZ-LP-P was 1.5-fold higher than of ACZ-LP (*p* < 0.001) and the maximum hypotensive effect resulted 1.4-fold higher (*p* < 0.001). The efficacy of acetazolamide significantly increased with the addition of the HPMC polymer. The confidence intervals for the difference of the means of the AUC_0–8h_ and the ΔIOP_max_ do not include the value of zero (Table 6).

As combination of the results obtained in stage 1 and stage 2 it can be observed that the formulation of ACZ in the hybrid liposome/HPMC system (ACZ-LP-P) produced an increment in ocular bioavailability (measured as AUC_0_**_–_**_8h_) 30.25-fold higher than that obtained when the drug was formulated as solution in the base vehicle (ACZ-VB).

### 3.3. In vivo Ocular Tolerance Studies

The ocular tolerance of the final prototype selected, ACZ-LP-P liposomal formulation, was in vivo tested according to the protocol described in materials and methods. Results are presented in Table 7.

For both formulations tested, rabbits’ eyes presented neither discomfort nor abnormal clinical signs during the six hours study in which a total of 12 instillations were performed. Furthermore, the cornea remained transparent throughout the test and the conjunctiva maintained its normal coloration. All the observations indicated an optimal tolerance for ACZ-LP and ACZ-LP-P formulations.

## 4. Discussion 

The tolerance of eye-drops intended for glaucoma treatment is a critical factor. Patients suffering this chronic disease are forced to instill the formulation several times per day. This frequent administration initially damages the precorneal film, a protective layer covering the ocular surface. This precorneal film is formed by an external lipid layer mainly composed of phospholipids, a subsequent aqueous layer in which mucins and enzymes among other components are dissolved, and finally a transmembrane mucin layer connecting this precorneal film with the corneal cells [48]. The alteration of this layer promotes the partial evaporation of the aqueous content and produces severe injury on corneal epithelium. It can even provoke “dry eye syndrome” [49]. It has been considered that preservatives included in multi-dose eye-drops formulations were the main cause of this important side effect. However, it is well known that the intrinsic nature of the active compound can also promote damage on the ocular surface; this is, for example, the case of timolol maleate [33]. Considering that, the formulation of antihypertensive drugs in eye-drops might be not only preservative-free but ideally might also include agents able to protect the ocular surface, such as osmoprotectants, antioxidants and phospholipids. 

The objective of this experimental work was then to create a hybrid formulation able to increase the bioavailability of active compounds with poor solubility on the ocular surface and, potentially at the same time, ensure the integrity of the precorneal film. With this in mind, liposomes were selected as suitable platform able to load low water-soluble compounds such as acetazolamide [50,51]. These systems are known to increase the retention time of several drugs on the ocular surface [52]. Trying to mimic the precorneal tear film, liposomes were prepared with phosphatidylcholine as the main component, a phospholipid widely present on the lipid layer, and hence demonstrated a very good tolerance profile on the ocular surface. In fact, an artificial tear containing several components similar to those present in the precorneal film has been recently proposed including phosphatidylcholine as lipid component [37,53]. Vitamin E, a hydrophobic antioxidant compound, was also included in the liposome formulation to improve their stability and to contribute to ocular surface protection. Furthermore, the ophthalmic composition included two osmoprotectants (trehalose and erythritol). The benefits of trehalose are based on its ability to protect cells from desiccation and restore damaged epithelial cells offering also some extent of anti-inflammatory activity [53]. Similarly, erythritol, has not only the ability to mitigate the effects of hyperosmolar stress, but also has been proposed as anti-inflammatory compound. Additionally, certain potential antioxidant activity has been also postulated for this compound, very useful in protecting ocular surface [54]. 

In the present work, the method for elaborating liposomes was modified by the substitution of chloroform or mixtures of chloroform: methanol, typically used in the preparation of liposomes, by ethanol, to create an initial organic phase in which the liposomes components were dissolved. This change in solvents allowed the solubilization of a higher amount of acetazolamide, which is practically insoluble in chloroform but slightly soluble in ethanol. Furthermore, this change avoids the use of halogenated class 2 solvents, categorized as probable human carcinogens agents and also ozone-depleting chemical [55]. In fact, class 3 solvents, such as ethanol, are preferred for pharmaceutical preparations according to ICH Q3C guidelines [56]. Thanks to this technological approach, the liposomal formulation so prepared contained acetazolamide in a final concentration of 1.4 mg/mL. It was found that 64% of the dose resulted entrapped in the liposome (bilayer and inner aqueous media). Several authors have observed lower acetazolamide entrapment in phosphatidylcholine liposomes prepared using chloroform [51] or chloroform: methanol [18]. The higher entrapment values observed in the present work might be related to the change of solvent previously mentioned. As demonstrated by Hathout et al. (2007) [18], a significant interaction of the drug with the lipid bilayer occurs. This evidence, supported with the low aqueous solubility of acetazolamide, makes it logical to assume that most of the drug might be dissolved in the lipid bilayer.

The ACZ-liposomal preparation was finally diluted 1:2 either with the base vehicle (borates, trehalose and erythritol solution) or with the polymeric solution in the base vehicle. This dilution was performed in order to better compare with the control composed by the drug dissolved in the base vehicle (solubility 0.7 mg/mL). For both liposomal formulations, pH and osmolarity resulted in the physiological range. The polymer selected, HPMC, is a non-charged polymer commonly used in the formulation of artificial tears at the concentration used in this work (0.3%) [57,58,59]. This polymer increases the viscosity of the formulation, and as consequence, the retention time of the formulation on the ocular surface [60], but in both cases remained in the range of natural tears. Surface tension values, also in the range of natural precorneal film values (43.6 ± 2.7 mN/m) [40], were low enough to enhance the spreading of the formulation on the ocular surface but not so low as to promote destabilization and damage on the precorneal film [61]. 

Tolerance of topical ophthalmic formulations in chronic therapies is a critical issue. Recent studies have shown that the inclusion in the ocular topical formulations of some polymers such as hyaluronic acid (HA), HPMC or carboxymethylcellulose (CMC) provided an additional positive effect improving the ocular tolerance of eye-drops [32]. More precisely, in the case of HPMC, it has been demonstrated that its inclusion in artificial tears intended for patients suffering from dry eye disease [53] or in antihypertensive formulations for glaucoma treatment [62] can increase the tolerance of the formulations. 

The inclusion of acetazolamide in the liposomal formulation prepared in this work increased its ocular bioavailability, according to AUC_0–8h_ measurements. Furthermore, its hypotensive activity appeared earlier, with a significant reduction in IOP in the first measurement time (1h) in comparison with acetazolamide solution, which needed 2 h to produce any effect. Interestingly, the effective time period was also increased, so at the end of the study, significant IOP reduction still appeared when the drug was formulated in liposomes although a tendency to recover normal IOP values was denoted.

There is still controversy concerning the right way to perform the in vivo antihypertensive studies. Some authors use, for example, the contralateral eye as control during the whole assay [18], other authors prefer to reserve an animal group for this purpose [51] or they use IOP basal (100%) data as reference [50], which is the option followed in this experimental work. Furthermore, the presentation of IOP data also differs from different works, in some cases the reduction is presented directly as “–X mmHg” while in other cases the data are showed as a percentage of IOP reduction. All these discrepancies in the experimental design and data presentation make difficult an absolute comparison between research works, thus only general tendencies are presented. Taking this into account, it might be worthy to comment that in the present work the same extent of IOP reduction than other previous works evaluating the hypotensive effect of acetazolamide loaded neutral liposomes was observed but with the administration of lower amounts of the drug. For example, Hathout et al. (2007) [8] instilled in each rabbit eye 50 uL of the liposomal suspension containing 1% of ACZ or El-Gazayerly and Hikal (1997) [51] administered 50 uL of the liposomal suspension containing 2% of the drug per eye, while in the present study only 25 uL of the liposomal suspension containing 0.07% of the antihypertensive drug was applied. 

The increment in drug ocular availability observed by the inclusion of acetazolamide in liposomes was even exacerbated when HPMC was combined with the liposomes. This final hybrid formulation produced an increase in acetazolamide ocular bioavailability more than 30 times, showing at the same time excellent tolerance behaviour in vivo. The new hybrid system proposed might reduce the administration frequency and also might increase the patient compliance. The strategy to combine nanosystems and hydrogels to improve the ophthalmic bioavailability of drugs after topical instillation has gained attention in the last years, which is the case of nanoparticles [63], nanoemulsion [64] or niosomes [65]. Furthermore, several research groups are also evaluating the combination of liposomes and hydrogels. For example, Yu et al., 2005 [66] prepared timolol maleate-loaded liposomes and included them in a gellan gum gel. This combination increased the drug effect period of time and reduced the onset time point. 

Among the polymers used to create the mentioned nano-hydrogel hybrid systems, Carbopol^®^, chitosan and natural gums have been the most commonly employed. However, other alternatives such as HPMC are being also studied. For example, Morsi et al., 2017 [64] prepared an acetazolamide-based nanoemulsion formulation by the inclusion of the combination of several polymers, HPMC among them, in the external phase. In agreement with our results, the authors observed that HPMC acted as a viscosity enhancer able to prolong the IOP lowering in glaucomatous rabbits this effect was attributed to the increase of the nanoemulsion retention time on the ocular surface. 

The mechanism by which the drug bioavailability after liposomal administration is increased, remains unclear; even more when they are included in a hydrogel. It has been established that vesicles can produce an intimate contact with corneal cells due to their surface charge (in the case of cationic liposomes) or to the presence of any bioadhesive or viscosizing agent (which is the case of HPMC) [50]. In this scenario, the high concentration of drug released in the vicinity of corneal epithelium might improve its passive diffusion trough corneal barrier. However, another hypothesis such as the penetration of liposomes itself into the corneal cells or even the modification of the tight-junctions of corneal epithelium by liposomes might not be completely excluded. In any case, further studies are necessary to investigate those hypotheses.

## 5. Conclusions

In the present study, the combination of acetazolamide-loaded liposomes and polymer solutions with osmoprotectants was evaluated as a new strategy to increase the drug bioavailability after ocular instillation. Materials used in the formulation were carefully selected to potentially ensure precorneal film integrity and in vivo ocular tolerance with optimal results. The in vivo activity study demonstrated that the new hybrid formulation proposed was able to promote sustained hypotensive effect for several hours, potentially reducing the administration frequency and increasing the patient compliance. The acetazolamide-liposome/HPMC/osmoprotectants hybrid system can be considered a promising topically applied dosage form for the treatment of glaucoma.

## 6. Patents

National Patent #2284398. Formulación de Vesículas Liposomales en Soluciones Acuosas con Características de Película Lagrimal. Available online: http://www.oepm.es/pdf/ES/0000/000/02/28/43/ES-2284398_B2.pdf accessed on 11 April 2018). 

## Figures and Tables

**Table 1:** Composition of liposomal formulations.

ACZ Formulation	Composition
ACZ-LP	Acetazolamide (0.7 mg/mL) and liposomes (expressed in concentration of PC, 20 mg/mL) in base vehicle (8.38‰ H_3_BO_3_, 0.755‰ Na_2_B_4_O_7_, 29.8‰ trehalose and 6.1‰ Erythritol).
ACZ-LP-P	Acetazolamide (0.7 mg/mL), liposomes (expressed in concentration PC, 20 mg/mL) and HPMC (0.3%) in base vehicle.

**Table 2:** Mean particle size, pH, surface tension, osmolarity, and viscosity data of the ACZ liposomal formulations. Data are expressed as means ± standard error.

	ACZ-LP	ACZ-LP-P
Size	157.3 ± 4.9	169.7 ± 6.1
pH	6.5 ± 0.1	7.0 ± 0.1
Surface tension (mN/m)	30.6 ± 0.9	47.6 ± 0.5
Osmolarity (mOsm/L)	297.7 ± 1.9	295.5 ± 1.5
Viscosity (mPa·s)	0.9 ± 0.1	4.7 ± 0.1

**Table 3:** Probability values obtained for the different sources of variation of the statistical model proposed.

Source of Variation	*P*-Value (AUC_0–8h_)	*P*-Value (ΔIOP_max_)
Treatment	<0.001	<0.001
Period	0.900	0.414
Animal	0.550	0.744
Eye (animal)	0.432	0.260
Variability explained by the model (%)	90.5	87.4
Degrees of freedom	20	20

**Table 4:** Stage one: mean values of area under the ΔIOP-time curve and the ΔIOP_max_ of each treatment (90% confidence limits).

Formulation	AUC_0–8h_ (%·h)	ΔIOP_max_ (%)
ACZ-LP	137.4 (122.4–152.5)	16.6 (14.9–18.4)
ACZ-VB	58.9 (40.1–77.8)	10.1 (8.6–12.1)
VB	7.2 (−0.2–14.6)	3.9 (2.4–5.4)

**Table 5:** Stage two: mean values of area under the ΔIOP-time curve and the ΔIOP_max_ of each treatment (90% confidence limits). For the purpose of comparison, the vehicle base data have been also included in Table 5.

Formulation	AUC_0–8h_ (%·h)	ΔIOP_max_ (%)
ACZ-LP	7.2 (−0.2–14.6)	3.9 (2.4–5.4)
ACZ-LP-P	142.6 (128.9–156.3)	18.0 (16.3–19.7)
VB	217.8 (203.8–231.8)	25.0 (23.9–26.1)

**Table 6:** Relationship between the two mean difference test (*P*-value of Student’s test) and confidence intervals for the difference of the means. Formulation ACZ-VB and formulation ACZ-LP.

	*P*-Value for H0: Difference = 0	95% Confidence Interval
Maximal IOP reduction (%)	<0.001	5.6 < F2 − F1 < 10.4
AUC_0–8h_ (%·h)	<0.001	58.2 < F2 − F1 < 98.8

**Table 7:** Macroscopic evaluation of signs and symptoms in the in vivo tolerance study for liposomal formulations.

Symptoms Studied	ACZ-LP	ACZ-LP-P
DISCOMFORT	Grade 0 (no reaction)	Grade 0 (no reaction)
CORNEA	Grade 0 (unaltered)	Grade 0 (unaltered)
CONJUNCTIVA	Grade 0 (unaltered)	Grade 0 (unaltered)
DISCHARGE	Grade 0 (no discharge)	Grade 0 (no discharge)
EYELIDS	Grade 0 (no swelling)	Grade 0 (no swelling)

## List of Abbreviations

HPMC	hydroxypropyl methylcellulose
ACZ-LP	acetazolamide-loaded liposomes, dispersed in the osmoprotectant solution
IOP	intraocular pressure
ACZ	acetazolamide
BCS	biopharmaceutical classification system
PC	phosphatidylcholine
Ch	cholesterol
LP	liposomes
VB	vehicle base
ACZ-LP-P	liposomal formulation included in the HPMC
EE	entrapment efficiency
ΔIOP	IOP pressure reduction
ΔIOPmax	maximum percentage of IOP reduction
AUC_0–8h_	area under the ΔIOP (%)-time curve from 0 to 8 h
*t*_onset_	the onset time of hypotensive effect
TBUT	fluorescein tear film breakup time
ACZ-VB	acetazolamide in base vehicle
HA	hyaluronic acid
CMC	carboxymethylcellulose
SLN	solid lipid nanoparticles
NLC	nano-structured lipid carriers
PLGA	poly lactic-co-glycolic acid
ANOVA	analysis of variance
